# Advances in the role of membrane-bound transcription factors in carcinogenesis and therapy

**DOI:** 10.1007/s12672-024-01414-1

**Published:** 2024-10-15

**Authors:** JiaLi Deng, Jie Zhou, BinYuan Jiang

**Affiliations:** 1 Medical Research Center, The Affiliated Changsha Central Hospital, Hengyang Medical School, University of South China, Changsha, 410004 China; 2 Department of Clinical Laboratory, The Affiliated Changsha Central Hospital, Hengyang Medical School, University of South China, Changsha, 410004 China

**Keywords:** Membrane-bound transcription factor, Nucleocytoplasmic shuttling, Carcinogenesis, Cancer therapy

## Abstract

Protein shuttling between the cytoplasm and nucleus is a unique phenomenon in eukaryotic organisms, integral to various cellular functions. Membrane-bound transcription factors (MTFs), a specialized class of nucleocytoplasmic shuttling proteins, are anchored to the cell membrane and enter the nucleus upon ligand binding to exert their transcriptional regulatory functions. MTFs are crucial in cellular signal transduction, and aberrant nucleocytoplasmic shuttling of MTFs is closely associated with tumor initiation, progression, and resistance to anticancer therapies. Studies have demonstrated that MTFs, such as human epidermal growth factor receptor (HER), fibroblast growth factor receptor (FGFR), β-catenin, Notch, insulin-like growth factor 1 receptor (IGF-1R), and insulin receptor (IR), play critical roles in tumorigenesis and cancer progression. Targeted therapies developed against HERs and FGFRs, among these MTFs, have yielded significant success in cancer treatment. However, the development of drug resistance remains a major challenge. As research on MTFs progress, it is anticipated that additional MTF-targeted therapies will be developed to enhance cancer treatment. In this review, we summarized recent advancements in the study of MTFs and their roles in carcinogenesis and therapy, aiming to provide valuable insights into the potential of targeting MTF pathways for the reseach of therapeutic strategies.

## Introduction

Eukaryotic organisms possess a nucleus enclosed by a double-layered nuclear membrane, which separates the nucleus from the cytoplasm, allowing gene expression and protein synthesis to occur in distinct spatial environments. This separation enables the precise regulation of cellular activities through the exchange of substances between the nucleus and the cytoplasm. The dynamic equilibrium of nucleocytoplasmic transport involves the translocation of various proteins and is a critical mechanism for cell cycle progression, acquisition of cellular functions, and differentiation of cell types [1, 2]. Disruption of this protein transport balance can lead to human disease, making nucleocytoplasmic transport a significant focus in tumor research as well. And altered nuclear localization of specific proteins, such as the nuclear translocation of tumor suppressor and oncogenic proteins, can lead to cellular transformation and cancer progression [3].

Membrane-bound transcription factors (MTFs) are proteins distributed across the cell membrane, cytoplasm, and nucleus. These proteins are synthesized and transported to the cell membrane, where they bind with ligands upon reception of external signals. Upon ligand binding, MTFs traverse the cytoplasm and enter the nucleus, where they function as transcription factors to drive the expression of relevant genes. This process enables biological responses to external signals and constitutes a crucial pathway for controlling gene expression in eukaryotes. Once inside the nucleus, MTFs influence various cellular processes such as apoptosis, proliferation, and migration [4, 5]. Aberrant nucleocytoplasmic shuttling of MTFs is frequently associated with the initiation and progression of malignant tumors. Activated genes following the nuclear entry of MTFs may weaken cell adhesion, enhance tumor cell migration and metastatic capabilities, and confer resistance to apoptosis. Investigating the molecular events involving MTFs aids in understanding tumor development and exploring relevant therapeutic approaches.

This review summarizes the significant and representative roles of membrane-bound transcription factors in tumor initiation and progression, as well as their potential in diagnosis and treatment. It is hoped that this review will provide insights for future research on MTFs in cancer therapy.

## The way of MTFs nuclear translocation

Nuclear translocation is a subcellular process whereby activated cytoplasmic proteins are transported into the cell nucleus to alter cellular functions [6]. Membrane-bound transcription factors are synthesized in the cytoplasm and swiftly transported to the cellular membrane, where they remain in a quiescent state as receptors. Upon exposure to specific environmental stimuli, these MTFs are activated through ligand binding, triggering their signaling functions. This activation prompts the translocation of MTFs from the membrane into the cytoplasm and eventually into the nucleus. Established signals for this nuclear translocation include ligand-receptor interactions as well as various stress conditions.

### The nuclear translocation route of MTFs

Current research categorizes the nuclear translocation pathways of membrane-bound transcription factors (MTFs) into three primary types. The first is the Golgi-ER retrograde trafficking route, where MTFs retain their full-length structure. These MTFs are endocytosed, relocated to the Golgi apparatus, and transported to the endoplasmic reticulum (ER) via COPI-coated vesicles before nuclear translocation. Epidermal Growth Factor Receptor (EGFR) exemplifies this pathway, and most of EGFR proteins were degraded by the lysosomal/proteasomal system or recycled back to the cell membrane for reuse. However, a small fraction of EGFR proteins translocated into the nucleus where they function as transcription factors. This nuclear EGFR is sufficient to drive the expression of downstream molecules, helping cells to cope with external stressors [7]. The second pathway is the Regulated Intramembrane Proteolysis (RIP) pathway, where MTFs are activated by external signals, recognized and cleaved by proteases, and their active fragments are released into the cytoplasm and subsequently translocated to the nucleus to regulate gene transcription. Notable MTFs following this pathway include Notch, Leukocyte-common antigen-related receptor tyrosine phosphatase (LAR), and amyloid precursor protein (APP) [8]. The third pathway involves MTFs anchored to the plasma membrane by lipid modifications; upon receiving stimuli, they undergo depalmitoylation, releasing them for nuclear translocation. Nuclear factor of activated T-cells 5, isoform α (NFAT5α), is a key example [9]. While these pathways are well-characterized, numerous aspects of MTF nuclear translocation remain poorly understood.

### The nuclear translocation mechanism of MTFs

The MTFs occurs through the nuclear pore complex (NPC), a basket-like structure embedded in the nuclear membrane. The NPC serves as a dual-function, bidirectional hydrophilic channel that controls the movement of substances into and out of the cell nucleus. It facilitates the import of shuttling proteins through both the central and peripheral channels simultaneously [10, 11]. Researchers categorize the process of protein nuclear import into two main pathways: the classical pathway and the non-classical pathway.

#### The classic nuclear translocation pathway of MTFs

**Fig. 1:**
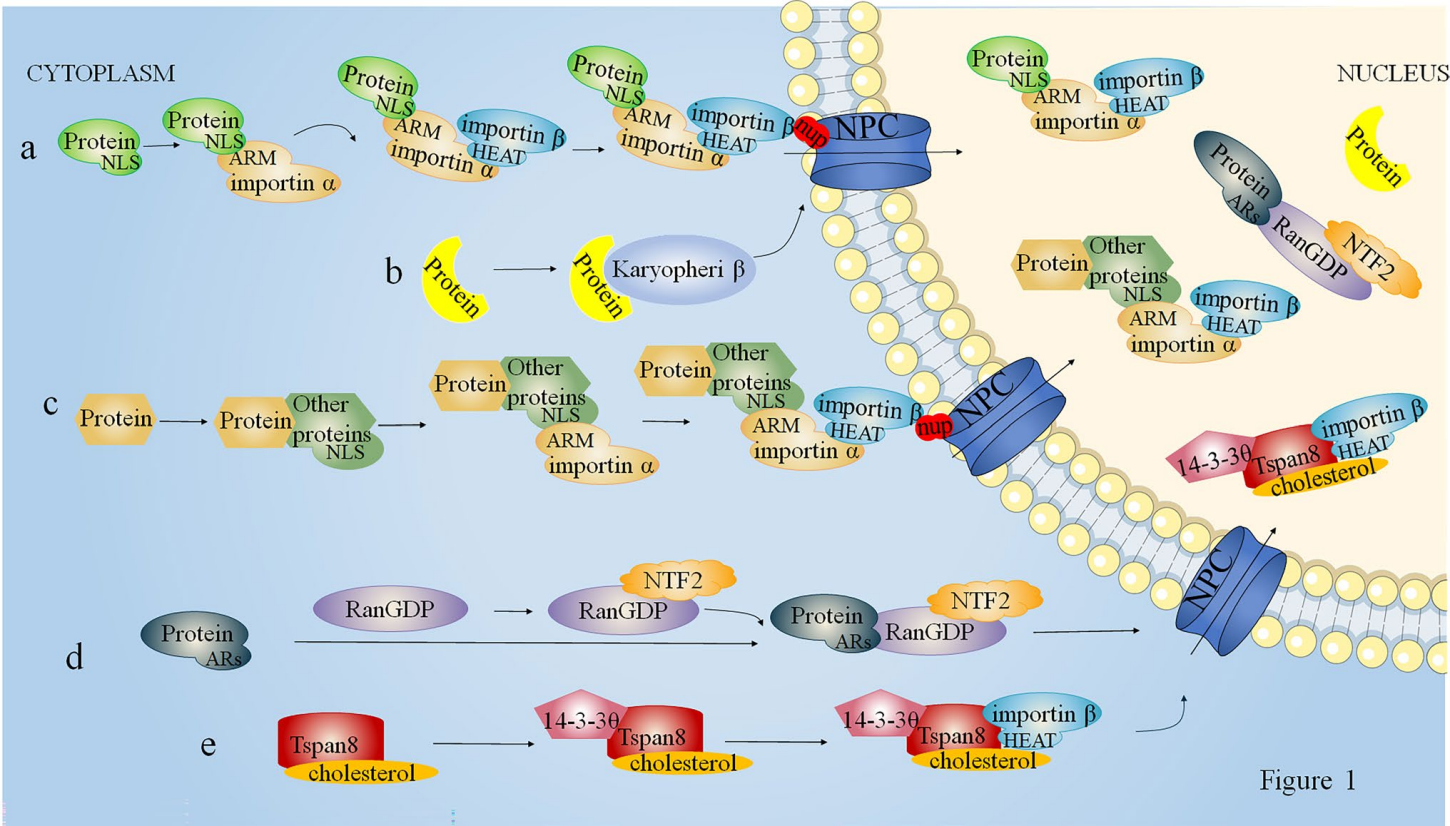
The process of MTFs transport into nuclear. **a**. Classic nuclear localization of MTFs; **b** MTFs enters the nucleus by interacting with Karyopherinβ; **c** MTFs attach to NLS-containing proteins to enter the nucleus; **d** The RaDAR nuclear import signal pathway; **e** Pathway of the 4 times transmembrane protein Tspan8 into the nucleus

The classical nuclear import pathway is the mechanism by which MTFs are transported into the cell nucleus through the interaction of importin α and importin β proteins [12]. The ARM structure of importin α recognizes the nuclear localization signal (NLS) presented by nucleocytoplasmic shuttling proteins, and its N-terminal structural domain recruits and binds to the HEAT structural domain of importin β. This forms a cargo protein-importin α-importin β complex. Importin β interacts with the FG repeat domains of nucleoporins (nups), facilitating the passage of the complex through the NPC into the nucleus. Ultimately, within the nucleus, Ran-GTP competitively binds to importin β, inducing a conformational change that leads to the dissociation of the shuttle protein-importin α-importin β complex, allowing the protein to remain in the nucleus (Fig. 1a). This classical pathway represents the predominant mechanism for the nuclear translocation of most proteins, though different proteins may utilize varied nuclear import mechanisms.

### The non-classic nuclear translocation pathway of MTFs

Protein nucleocytoplasmic shuttling is a crucial pathway for intra- and intercellular communication, enabling the bidirectional transmission of information through this mechanism. Approximately 50% of nuclear proteins in human cells lack predictable nuclear localization signals (NLS) [13], necessitating alternative nuclear import pathways for many cytoplasmic proteins. For example, Nkx2-2 interacts with importin β1 and importin 13 to enter the nucleus, illustrating a non-classical nuclear import pathway involving direct interaction with Karyopherin β proteins [14, 15] (Fig. 1, b). Additional nuclear import mechanisms include a "piggy back" that attaches to proteins containing NLS for nuclear input [16] (Fig. 1, c); proteins containing ankyrin repeats (ARs), which bind directly to RanGDP, form a protein complex through RanGDP with nuclear transport factor 2 (NTF2), allowing it to enter the nucleus, RanGDP-ankyrin repeats nuclear import pathway [17] (Fig. 1d). In 2021, Yuwei Huang et al. [18] discovered that the palmitoylation-modified transmembrane protein Tspan8 forms a complex with cholesterol in the cytoplasm, binds to 14–3-3θ/importin-β, and translocates through the nuclear pore complex (NPC) into the nucleus(Fig. 1e). This finding revealed, for the first time, the possibility of various transmembrane proteins entering the nucleus to perform functions.

## Representative membrane-bound transcription factors in human

MTFs are widely observed in eukaryotic organisms, and research indicates their significant involvement in human growth, development, and disease. In this review, we will focus on several typical MTFs associated with human tumors, summarizing their functions (Table 1) and discussing recent findings related to their roles in tumorigenesis and cancer therapy.

**Table 1:** Functions, Targets and Inhibitors of MTFs

MTF	Function	Target	Inhibitor
EGFR	Regulation of gene transcription	E2F1-mediated upregulation of *POMC* expression [19]	Osimertinib [20], BLU-945 [21], EAI045 [22]
		STAT3-mediated activating the promoter of *iNOS, CCND1, c-Fos, COX-2, SATA1, MMP-2* [23]	
		STAT5-mediated upregulation of *AURKA* expression [24]	
	Promote DNA replication and repair	PCNA stabilisation and PCNA- mediated chromatin complexes repair [25]	
HER2	Regulation of gene transcription	HAS-mediated activates transcription of *COX-2* [26] and inhibits transcription of *DEPTOR* [27]	Trastuzumab, Pertuzumab, Lapatinib, Naratinib, and T-DM1 [28]
		STAT3-mediated upregulate of *STAT1* and *CCND1* transcription and microRNA 21 co-activate [29]	
		RNA Pol I-mediated increased ribosomal RNA transcription for translation and cell growth [30]	
FGFR	Regulation of gene transcription	CREB-binds RSK1 and chromatin, recruits RNA Pol II and promotes the expression of *Jun, CCND1* and *FGF2* [31]	Pemigatinib [32], Futibatini [33], Infigratinib [34], Erdafitinib [35]
		ERα-inhibition of ERα binding to target gene promoters [36]	
		HIF-1α and HIF-2α- downregulation of HIF and p300 complex-mediated gene transcription [37]	
β-catenin	Regulation of gene transcription	TCF/LEF-upregulation of β-catenin downstream target expression affects Wnt transcriptional activity) [38]	Aspirin, indomethacin and curcumin [39]
	Promote cell proliferation	HIF1α and HIF2α-enhanced tolerance of tumour cells to hypoxia [40–42]	
Notch	Regulation of gene transcription	RUNX1-upregulation of IL7R gene expression [43]	
		p300-increases Notch target gene expression by acetylating H3K27 [44]	
IGF1R	Regulation of gene transcription and cell proliferation	RNA Pol II-binds chromatin, promotes the expression of *Jun* and *FAM21*; promote cell survival and migration[45]	Picropodophyllin and Linsitinib [46]
	Promote DNA replication and repair	PCNA-phosphorylates PCNA to increase cellular tolerance to DNA damage [47]	
IR	Regulation of gene transcription	HCF-1-enriched at gene promoters, regulates insulin-related functions and insulin resistance-related genes [48]	XMetA, XMetS, IRAB-A and XMetD [49]

### Human epidermal growth factor receptor

#### Function of HERs

The human epidermal growth factor receptor (EGFR) family consists of four members that belong to the erythroblastic leukemia viral oncogene homologue (ErbB) lineage of proteins: EGFR (HER1), ErbB2 (HER2), ErbB3 (HER3), and ErbB4 (HER4). Throughout this review, these kinases shall be referred to as EGFR, HER2, HER3, and HER4. These HERs are glycoprotein that belong to the receptor tyrosine kinase (RTK) family and span the cytoplasmic membrane. Its structure is primarily composed of three parts: an extracellular ligand-binding domain, a hydrophobic transmembrane domain, and an intracellular tyrosine kinase activity domain. HERs is predominantly activated by ligand binding to its extracellular domain, a cascade of downstream signaling pathways is initiated, which is critical for cell growth, migration, proliferation, and differentiation [50]. EGFR and HER2 are the most extensively studied members in the family, and this review will primarily focus on these two receptors.

**Fig. 2:**
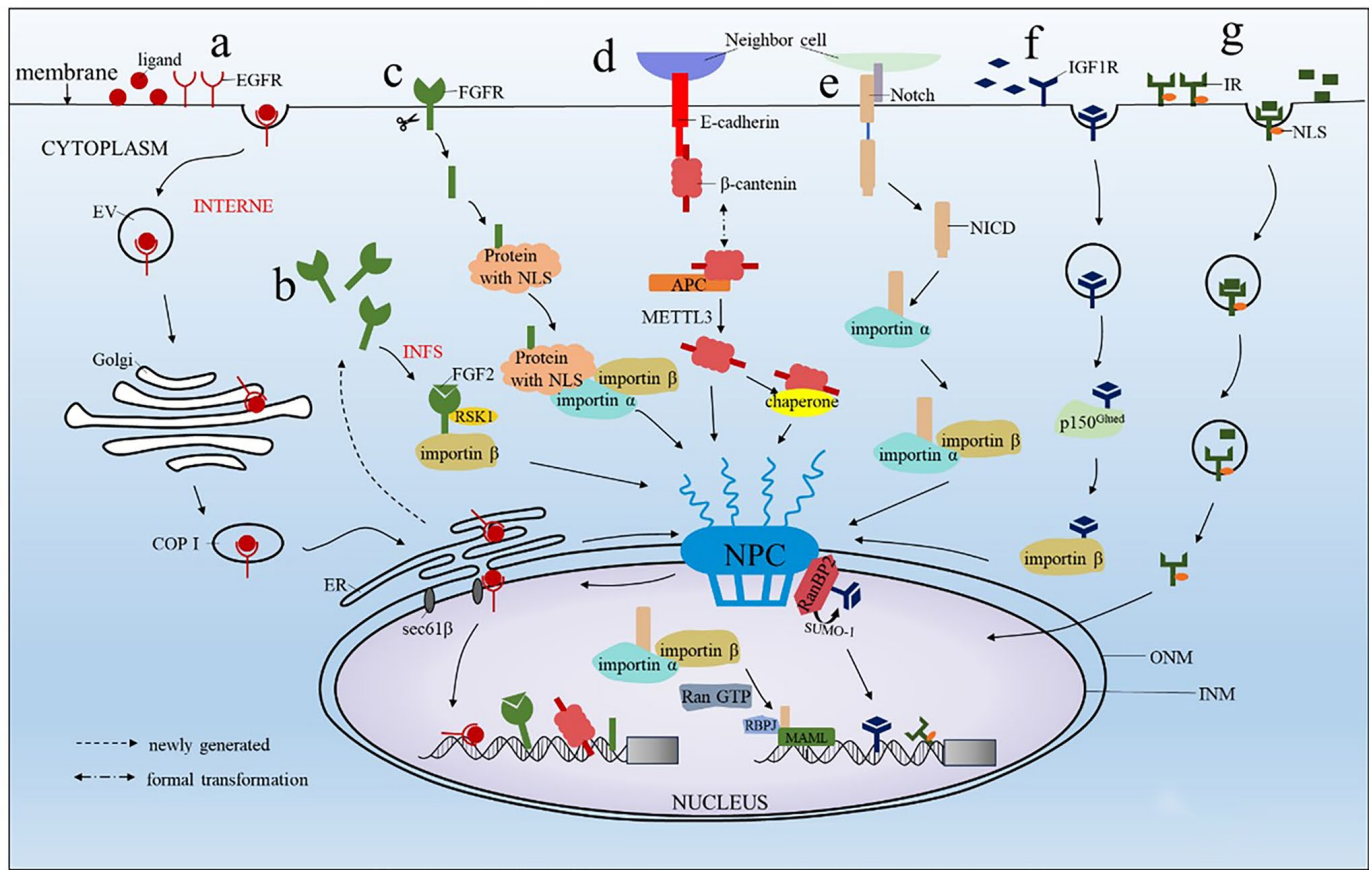
Schematic of typical MTF nuclear entry pathways following external stimuli. **a** EGFR nuclear entry process: endocytosed EGFR is transported to the nucleus via the Golgi, ER, and nuclear pore complex; **b** Cytoplasmic FGFR nuclear entry process: cytoplasmic FGFR binds to RSK1 and is transported into the nucleus by importin proteins; **c** Membrane-bound FGFR nuclear entry process: cleaved membrane-bound FGFRs interact with NLS-containing proteins and are transported into the nucleus by importin proteins; **d** β-Catenin nuclear entry process: undegraded β-catenin in the cytoplasm binds to its chaperones to facilitate nuclear entry; **e** NOTCH nuclear entry process: the cleaved Notch intracellular domain (NICD) is transported into the nucleus by importin proteins; **f** IGF1R nuclear entry process: SUMOylated IGF1R is transported into the nucleus by importin proteins; **g** IR nuclear entry process: activated IR is transported into the nucleus via an NLS-related pathway

Membrane-localised EGFR binds to the ligand and enters the cytoplasm via cytosis, after which part of the EGFR is transported to the early endosome, where it can be recycled back into the plasma membrane or transported to the lysosome for degradation, and a few enter the nucleus for signaling. Both EGFR and ErbB2 enter the nucleus via the INTERNET pathway[7]. Upon ligand binding, membrane-localized EGFR undergoes endocytosis into the cytoplasm and is trafficked to the Golgi apparatus. Through coat protein complex I (COP I)-mediated retrograde transport, EGFR reaches the endoplasmic reticulum and subsequently translocates to the inner nuclear membrane (INM) via the outer nuclear membrane (OMN) and nuclear pore complex (NPC) (Fig. 2, a). This pathway involves the endoplasmic reticulum-associated degradation (ERAD) system, where receptors are retrogradely translocated from the Golgi to the endoplasmic and carried into the cytoplasm [51]. This mechanism detects misfolded proteins in the endoplasmic reticulum and carries them to the cytoplasm, where they are ubiquitinated and degraded, and also assists the entry of transmembrane proteins into the nucleus. Once at the INM, EGFR associates with the translocase sec61β, enabling its release from the nuclear membrane into the nucleoplasm [52, 53].

EGFR has no DNA-binding domain, so after entering the nucleus, it mainly plays a transcriptional regulatory role by binding to other DNA-binding proteins or transcription factors. By binding to its targets E2F1, STAT3 and STAT5, nuclear EGFR can regulate the expression of many genes, including *iNOS*, *CCND1* (cyclin D1), *c-Fos*, *PTGs 2* (COX-2), *SATA1*, *MMP-2*, *AURKA* (Aurora A) and *MYBL2* (eB-Myb) [19, 23, 24]. Besides, nuclear EGFR can also phosphorylate proliferating cell nuclear antigen (PCNA), stabilize PCNA-chromatin complexes, and promote cell proliferation and DNA mismatch repair [25] (Table 1). This suggests a potential mechanism by which nuclear EGFR functions as a transcriptional activation factor to regulate gene expression.

HER2 is overexpressed in 13–22% of breast cancers (BC), with 60–70% of these cases also co-expressing hormone receptors (HRs). Once HER2 translocates to the nucleus, it can directly bind to the HAS (HER-2-associated sequence) site of DNA promoters, activating the transcription of *COX-2* [26] and inhibiting the transcription of DEPTOR (DEP-domain containing mTOR interaction protein) [27]. Additionally, HER2 in the nucleus can interact with STAT3, thereby regulating the transcription of *STAT1* and *CCND1* and acting as a coactivator of microRNA 21 (miR-21) in breast cancer, revealing a novel function of nuclear ErbB-2 as a regulator of microRNAs expression [29]. Nuclear HER2 also binds to RNA polymerase I (RNA Pol I) and β-actin, enhancing the association of RNA Pol I with ribosomal DNA, which increases ribosomal RNA transcription, promoting translation and cell growth [30] (Table 1).

#### HERs in carcinogenesis and therapy

Drugs targeting EGFR have achieved remarkable success in cancer treatment. Third-generation EGFR inhibitors, represented by Osimertinib, have saved the lives of countless cancer patients and have been approved by major regulatory agencies for the treatment of EGFR T790M-positive cancer [20], However, Osimertinib resistance is surfacing as treatment is rolled out. To overcome these resistance mutations, a fourth-generation EGFR inhibitor is being developed and is undergoing preclinical evaluation[10]. BLU-945 is one of the fastest advancing fourth-generation EGFR inhibitors in research, and BLU-945 alone or in combination with oxitinib showed early signals of clinical activity and was well tolerated in a large number of pre-treated EGFR-mutated non-small cell lung cancer patients[21]. The combination of EAI045 with cetuximab shows desirable tumour-suppressive effects in both in vivo and ex vivo experiments [22].

Several HER2 inhibitors such as Trastuzumab, Pertuzumab, Lapatinib, Naratinib, and Trastuzumab emtansine (T-DM1) have been approved for the treatment of HER2-positive breast cancer [28]. The interaction between HER2 and HR pathways complicates responses to both anti-HER2 and endocrine therapies. Dual receptor blockade (HER2 and ER) may overcome therapy resistance, reduce chemotherapy reliance, and improve survival outcomes while maintaining safety and tolerability [54]. Activating mutations in HER2 and co-occurring HER3 mutations enhance oncogenesis and invasion via PI3K activation, complicating treatment by reducing sensitivity to HER2 inhibitors [55].

The research of targeting nuclear HERs is becoming the focus. Abnormal nuclear accumulation of HERs is implicated in cancer progression, adverse prognosis, and increased resistance to therapy [56]. It serves as a crucial diagnostic marker and therapeutic target for various tumors, especially EGFR and HER2 altering in lung cancer and HER2 altering in breast cancer [57]. Patients with high levels of nuclear EGFR in cancer often presented poorer clinical outcomes, associated with resistance to cetuximab and gefitinib [58]. Her2 has also been shown to be associated with paclitaxel resistance in breast cancer [59].

Researchers found the inhibition of EGFR nuclear transport can enhance the sensitivity of triple-negative breast cancer cells to cetuximab, providing a promising treatment strategy for this cancer subtype [58]. Moreover, EGFR translocation to the nucleus has been observed following treatments such as cisplatin or radiotherapy [60]. As resistance develops, clinical treatment outcomes are often limited. Studies have demonstrated that overexpression of TIP30 can restore the sensitivity of non-small cell lung cancer cells to gefitinib by attenuating both cytoplasmic and nuclear EGFR signaling pathways [61]. Targeting intact EGFR and PKM2 complexes in the nucleus may overcome resistance to cancer radiotherapy [62]. Furthermore, Siyoung Ha et al. [63] identified a small organic compound, a 2-aminothiazole derivative, which inhibits the nuclear translocation of EGFR by competitively binding to importin β1, thereby blocking its interaction with EGFR in a non-classical pathway. This compound significantly inhibited the invasion of lung cancer H1299 cells without affecting EGFR receptor function.

In summary, elevated levels of nuclear EGFR are associated with inferior clinical outcomes in certain cancer types. Strategies aimed at inhibiting EGFR nuclear translocation hold promise for improving overall survival rates, underscoring the importance of targeting intracellular trafficking pathways of EGFR in cancer treatment.

### Fibroblast growth factor receptor

#### Function of FGFRs

The Fibroblast Growth Factor Receptor (FGFR) is a transmembrane glycoprotein activated through interaction with its natural ligand, Fibroblast Growth Factor (FGF). FGFR comprises three extracellular immunoglobulin-like domains responsible for binding with FGF, a transmembrane helical domain, and an intracellular domain [64]. To date, five distinct membrane-bound FGFRs have been identified. Among these, FGFR1-4 exhibit tyrosine kinase activity, whereas FGFR5 (also known as FGFRL1) can only serve as an FGF receptor but lacks an intracellular domain and tyrosine kinase activity, thereby unable to regulate signal transduction [65]. FGFR plays a pivotal role in various cellular processes, including the promotion of angiogenesis, limb growth, and the growth and differentiation of diverse cell types [66].

FGFR is synthesised and released directly into the cytoplasm or transported to the surface of the cell membrane for localisation, with a few entering the nucleus for signaling. Three primary mechanisms are under investigation for the nuclear entry of FGFRs. The first mechanism involves full-length membrane-bound FGFRs entering the nucleus via the INTERNET pathway, akin to EGFR [67]. The second mechanism entails FGFRs synthesized in the nucleus being released directly into the cytoplasm or released from endoplasmic reticulum vesicles through the Sec61 channel. In this pathway, soluble FGFR1 interacts with the ligand FGF2 and the nuclear ribosomal S6 kinase A1 (RSK1) containing a NLS, subsequently being imported into the nucleus via importin β. This pathway is termed the integrative nuclear FGFR1 signaling model (INFS) [68] (Fig. 2, b). The third mechanism involves the cleavage pathway of FGFR, where proteases such as the proteasome and γ-secretase cleave FGFR1 and FGFR3 into smaller truncated variants [69].

These variants stabilize in the cytoplasm by binding to other proteins containing NLS and enter the nucleus depending on importin α and importin β [31] (Fig. 2, c). Once in the nucleus, FGFR can function as a transcription factor to regulate gene expression. Previous studies have shown that nuclear FGFR1 binds to chromatin by interacting with CREB-binding protein and RSK1, and further recruits RNA polymerase II (RNA Pol II) for gene transcriptional regulation, which promotes the expression of *Jun*, *CCND1*, *FGF2* [31] (Table 1).

#### FGFRs in carcinogenesis and therapy

Nuclear FGFR is also associated with cancer. Different FGFR subtypes exhibit distinct associations with cancer. Nuclear FGFR1 has been implicated in promoting the proliferation and invasion of pancreatic cancer cells [70], activating the transcription of genes crucial for the migration of breast cancer cells, and driving the expression of genes associated with resistance to anti-estrogen therapy in ER + breast cancer, contributing to poor patient prognosis [71]. In ER-expressing breast cancers, nFGFR1 interacts with ERα and reduces binding of ERα to its target gene promoters [36]. FGFR2 functions as a metastasis suppressor by regulating hypoxia-inducible factor-mediated hypoxic response, thereby attenuating the invasion of prostate cancer cells [37]. However, nuclear FGFR2 is often associated with increased tumor size and lower overall survival rates in various types of breast cancer [72]. However, nuclear FGFR2 is often associated with increased tumor size and lower overall survival rates in various types of breast cancer [73]. FGFR3 is highly expressed in the nucleus of pancreatic cancer cells and is positively correlated with high N stage and poor prognosis [74], although its specific targets remain unknown. In summary, nuclear FGFR is closely linked to cancer proliferation, invasion, drug resistance, and prognosis. Consequently, targeting the nuclear translocation of FGFR is emerging as a promising avenue in cancer therapy.

Multiple FGFR inhibitors are being investigated in clinical trials, four inhibitors have been approved by the FDA for the treatment of tumors. Pemigatinib is approved for the treatment of locally advanced or metastatic bile duct cancer [32]. Futibatini is approved for the treatment of intrahepatic bile duct cancer [33]. Infigratinib is a first-line treatment for advanced or inoperable bile duct cancer [34]. Erdafitinib is indicated for the treatment of metastatic urothelial carcinoma, as well as urothelial bladder cancer with FGFR2 or 3 mutations [35]. In addition, a number of FGFR inhibitors are currently being studied in phase II trials to better treat cholangiocarcinoma and urothelial carcinoma.

### β-catenin

#### Function of β-catenin

Β-catenin is the central molecule of the canonical Wnt signaling pathway, primarily responsible for transmitting signals to the cell nucleus and playing a crucial role in physiological homeostasis [75]. It is mainly anchored to the cell membrane by calcium-binding protein-E (E-cadherin, E-cad), with a small amount found freely in the cytoplasm [76]. Its abnormal overexpression is implicated in various diseases, including cancer. The β-catenin protein consists of twelve Armadillo repeat sequences (R1-R12) forming a rigid central region, flanked by flexible amino-terminal domain (NTD) and carboxy-terminal domain (CTD). Between the last Armadillo repeat sequence and the flexible region of the CTD lies the conserved Helix-C, which determines β-catenin's ability to serve as a scaffold, anchoring ligand proteins in the cytoplasm, cell membrane, or nucleus [77].

Under the protection of Adenoma Polyposis Coli (APC) protein in colorectal adenomatous polyposis, β-catenin can remain in the cytoplasm without degradation. Upon activation of the Wnt signaling pathway, it translocates to the cell nucleus, regulating gene expression. The shuttling of β-catenin between the nucleus and cytoplasm is also considered a critical feature of Wnt canonical pathway activation [78]. β-catenin has been shown to lack a NLS and enters the nucleus by directly binding to the nuclear pore, without requiring assistance from other transport proteins [79]. Additionally, β-catenin can bind to some chaperones such as Smad3/4, FOXM1, MUC1, IRS1, BCL9, and LEF1 to facilitate nuclear entry [80]. Recent studies have found that METTL3 can promote β-catenin nuclear translocation by downregulating c-Met kinase, thereby inhibiting the interaction between β-catenin and E-cadherin and its membrane localization (Fig. 2, d) [81]. However, β-catenin lacks DNA binding ability. Upon entering the nucleus, it forms a transcriptional complex with T cell factor/lymphoid enhancer factor (TCF/LEF), acting as a transcriptional activator, activating the expression of downstream targets of β-catenin, and influencing Wnt transcriptional activity (Table 1) [38].

#### β-catenin in carcinogenesis and therapy

Directly targeting β-catenin to block Wnt signaling is an effective way to treat cancer. Aspirin and indomethacin have been found to down-regulate the transcriptional activity of β-catenin/ TCF response genes, and Phase 3 trials are underway in which low-dose aspirin therapy leads to reduced levels of biomarkers associated with colorectal cancer risk. The safety and efficacy of curcumin in combination with 5-fluorouracil to inhibit β-catenin in patients with metastatic colon cancer are also in Phase I trials [39].

Nuclear β-catenin is intricately involved in the onset and progression of cancer. It has been reported that β-catenin can directly bind to HIF1α and HIF2, promote their transcription, and enhance the tolerance of tumor cells to hypoxia, which promotes the proliferation of pancreatic cancer, hepatocellular carcinoma, gastric cancer, and other types of cancer cells and therapeutic resistance in colorectal cancer [40–42]. Several indirect pathways targeting β-catenin to achieve anticancer purposes have been identified. Chunhua Wan et al. [82] found that histone lysine methyltransferase KMT2A is a key participant in β-catenin-mediated transcription. Targeting KMT2A can weaken the binding of β-catenin to DNA motifs and its transcriptional activity, reducing the malignant potential of colorectal cancer (CRC). Study finds strong relationship between RUNX3 and β-catenin [83]. It has been discovered that RUNX3 can competitively bind to β-catenin, preventing its nuclear entry, inhibiting classical Wnt signaling, thereby reducing the proliferation and invasion of gastric cancer, colorectal cancer and glioma, and promoting apoptosis [84–86]. The tumor suppressor Leucine zipper transcription factor-like 1 (LZTFL1) in gastric cancer can also inhibit β-catenin nuclear translocation to suppress the metastasis of gastric cancer cells [87]. Similarly, blocking β-catenin nuclear translocation can inhibit pancreatic tumor growth by disrupting the nuclear β-catenin/TCF1 complex [88].

Therapeutic approaches targeting β-catenin nuclear translocation is a promising potential strategy to inhibit tumor growth [89]. It has been found that lycopene can be used to promote apoptosis of gastric cancer cells by blocking the nuclear transport of β-catenin, which is expected to become a target drug for the treatment of gastric cancer [90]. Recently, Aidi Gao et al. [91] found that that TAB182, a binding protein of tankyrase 1, aggravates the progression of esophageal squamous cell carcinoma through the promotion of nuclear translocation of β-catenin, so that targeting the nuclear translocation of β-catenin can also become a novel therapeutic approach for esophageal cancer.

In summary, nuclear β-catenin can be considered a pro-carcinogenic factor, and reducing nuclear β-catenin expression holds promise for inhibiting tumor initiation and cancer progression. However, the development of β-catenin-targeted drugs still requires further exploration due to the limited drugability of current medications.

### Notch

#### Function of notch

Notch itself is a cell surface receptor composed of functional extracellular domains, transmembrane domains, and a Notch intracellular domain (NICD). It mediates short-range signaling by interacting with transmembrane ligands Delta and Jagged on adjacent cells, thereby regulating cell proliferation, differentiation, and apoptosis-related cellular processes. It is a highly conserved signaling pathway [92]. Both Notch receptors and ligands are single transmembrane proteins. Ligand binding leads to cleavage and release of NICD, which enters the nucleus via the classical nuclear import pathway (Fig. 2, e) [93]. Inside the nucleus, importin β binds to RanGTP to dissociate the complex. NICD assembles with DNA-binding protein RBPJ and Mastermind-like 1 (MAML1) to form the Notch transcriptional activation complex (NTC), acting as a transcriptional effector to activate the expression of Notch response genes [94]. After NTC assembly, binding to the enhancer at the distal end of RUNX1 regulates the expression of the interleukin-7 receptor (IL7R) gene [43], and can also increase Notch target gene expression by recruiting p300, acetylated histone H3K27 (Table 1).

#### Notch in carcinogenesis and therapy

The nuclear accumulation of NICD is closely associated with cell cycle regulation and proliferation during tumorigenesis, making it an important target for cancer-targeted therapy [95]. Research has found that long-term consumption of aspartame increases nuclear accumulation of NICD, enhances the enrichment of tumor stem cells, and promotes the invasiveness of pancreatic cancer cells (PANC-1), leading to malignant progression [96]. Additionally, nuclear accumulation of NICD promotes the development of intrahepatic cholangiocarcinoma arising from bile duct obstruction [97]. However, in cell lines lacking *Rb*, *p107*, and *p130*, overexpression of NICD1 protects against tumor growth [98]. In the therapeutic context of cancer, sclareolide can re-sensitize gemcitabine-resistant human pancreatic cancer cells (HPCCs) by mediating NICD [99]. Furthermore, diosgenin can prevent atherosclerosis by inhibiting the nuclear translocation of NICD [100]. Studies have shown that NICD promotes apoptosis and inhibits proliferation in pheochromocytoma cells, while stimulating the expression of autophagy-related proteins in PC12 cells [101].

However, recent research published in Cell indicates that nuclear accumulation of NICD in Drosophila follicle cells promotes tumor growth, reduces apoptosis, increases nuclear size, decreases DNA damage, and upregulates *raptor*, a conserved cell growth and metabolism gene, facilitating DNA damage repair and inhibiting apoptosis, thereby promoting cancer development [102]. These differences suggest that NICD may act differently in various cells or cancers, serving as either an oncogene or tumor suppressor in different tumor subtypes. Therefore, targeted approaches using Notch as a cancer therapy target should be tailored based on specific cellular effects.

### Insulin-like growth factor 1 receptor

#### Function of IGF1R

The Insulin-like Growth Factor 1 receptor (IGF1R) is located on the surface of human cells and belongs to a large class of tyrosine kinase receptors. It is activated by Insulin-like Growth Factor 1 (IGF-1) and Insulin-like Growth Factor 2 (IGF-2) [103]. Upon ligand binding, the tyrosine kinase domain of the receptor is activated. Subsequently, the binding site of the receptor is phosphorylated, initiating signal transduction that mediates growth, development, and adult synthetic metabolism, leading to excessive proliferation in skeletal muscle or other target tissues [104]. High level of IGF1R gene expression is a typical marker for most cancer types, and the anti-apoptotic and pro-cell survival capabilities of IGF1R may contribute to its role in cancer cells [105, 106].

A substantial body of evidence indicates the presence of nuclear IGF1R, which correlates with the transcriptional activity of the genome. Upon internalization, IGF1R enters the cytoplasm and is transported to the NPC by binding to the motor protein subunit p150^Glue^. It undergoes co-localization with importin β and subsequently binds to the nuclear pore protein RanBP2 to enter the nucleus. During this process, IGF1R undergoes sumoylation by Small Ubiquitin-like Modifier 1 (SUMO-1), which is necessary for its interaction with RanBP2. Conversely, RanBP2 can act as a SUMO E3 ligase, enhancing the sumoylation and stability of nuclear IGF1R, thereby promoting its nuclear accumulation (Fig. 2, f) [107].Nuclear IGF1R phosphorylates the Y60, Y133 and Y250 sites of PCNA, upregulates DNA damage repair, promotes PCNA ubiquitination and allows cells to evade G1 blockade [47] (Table 1).

#### IGF1R in carcinogenesis and therapy

IGF1R inhibitors Picropodophyllin (PPP) and Linsitinib can inhibit autophagy in human triple-negative breast cancer and improve the efficacy of chemotherapy immunotherapy in mice, which is a new strategy for cancer treatment in the context of chemical immunotherapy [46] (Table 1). The nuclear activity of IGF1R is prominent in cancer cells and often correlates with adverse clinical outcomes and tumor biological characteristics. Higher levels of nuclear IGF1R are significantly associated with shorter survival times and poor prognosis in patients [45]. In renal cell carcinoma, embryonal rhabdomyosarcoma, and synovial sarcoma, nuclear IGF1R in patients is associated with poor prognosis and tumor progression. It is also considered a marker of overall survival and progression-free survival in patients with soft tissue sarcoma and osteosarcoma undergoing anti-IGF1R antibody therapy [108]. In malignant prostate epithelium, IGF1R is induced to recruit to chromatin, directly binding to DNA and interacting with RNA Pol II to upregulate the expression of JUN and FAM21, thus participating in tumor cell survival and migration [45].

Regarding cancer therapy, higher concentrations of nuclear IGF1R are not only correlated with lower survival rates in metastatic colon cancer patients, but also observed in drug-resistant colon cancer cell lines. Moreover, the nuclear accumulation of IGF1R is also associated with resistance to chemotherapy and radiotherapy [109]. Additionally, reducing nuclear IGF1R enhances the DNA damage and G2/M arrest induced by Eribulin, overcoming resistance to Eribulin in colorectal cancer and significantly improving treatment outcomes [110]. Such evidence indicates the potential role of nuclear IGF1R in developing resistance to targeted therapy or other treatments. Recent research shows that nuclear localization of IGF1R leads to higher cell proliferation rates and earlier tumor development in pediatric gliomas, while cells become sensitive to OSI906 treatment, providing a new therapeutic direction for patients with gliomas with nuclear IGF1R localization [111]. Nuclear IGF1R can rescue damaged DNA replication forks, increase cell tolerance to DNA damage, and promote tumor cell proliferation, which may be the mechanism by which nuclear IGF1R promotes tumor development [112]. IGF1R-targeted inhibitors have also been under active development in recent years, but all of them have ended in failure due to their toxicity or inefficacy [113], so drugs targeting the IGF1R to treat cancer remain to be explored.

### Insulin Receptor

#### Function of IRs

The insulin receptor (IR) and IGF1R have evolved from a common receptor, and although these two receptors have similar structures, they play different roles. IR is a transmembrane tyrosine kinase receptor composed of 2 extracellular α subunits and 2 transmembrane β subunits, which can be activated by insulin from the pancreas, IGF-I, and IGF-II [114]. The interaction between IR and its activating ligands triggers tyrosine autophosphorylation of the β subunits, which is involved in the translocation of GLUT4 to the plasma membrane, glucose influx, glycogen synthesis, glycolysis, and fatty acid biosynthesis, and is also associated with neurodegenerative diseases and cancer [115].

Insulin binds to the IR and is rapidly internalised, and as they move towards the Golgi, insulin separates from the IR and is degraded in the nuclear endosomes as the acidic environment increases, whereas the IR can be recycled back to the plasma membrane or translocated to the lysosome for degradation, with a small amount entering the nucleus [116]. Kesten et al.'s latest research has identified potential NLS on IR and demonstrated that NLS is essential for insulin-induced nuclear translocation of IR (Fig. 2, g), but the specific mechanism of nuclear entry requires further exploration [117].

IR enters the nucleus in its full-length form, initially associates with RNA Pol II, inducing over-phosphorylation of its C-terminal domain (CTD). Subsequently, it is mediated by a transcription co-regulator called Host cell factor-1 (HCF-1), highly enriched on gene promoters, acting as a transcription factor regulating insulin-related functions and genes associated with insulin resistance [48] (Table 1). During this process, insulin not only promotes nuclear translocation of IR but also increases IR association with RNA Pol II, suggesting that insulin treatment increases recruitment of Pol II and IR binding at transcription start sites, providing new insights into the mechanism by which insulin treatment lowers blood glucose levels.

#### IRs in carcinogenesis and therapy

Recent studies have highlighted a significant association between the nuclear translocation of IR and the onset and progression of cancer. Under insulin treatment, IR can translocate into the nuclei of lung cancer cells, thereby promoting cell proliferation [118]. Fructose-1,6-bisphosphate aldolase (ALDOB) interacts with IR and inhibits its nuclear translocation. In hepatocellular carcinoma, downregulation of ALDOB enhances IR signaling, contributing to the development and poor prognosis of the disease [119]. These findings suggest that targeting the nuclear translocation of IR may offer therapeutic benefits for liver cancer. Research into anti-nuclear IR antibodies has revealed two types: agonistic and antagonistic. Agonistic antibodies, including XMetA, XMetS, and IRAB-A, have been shown to significantly improve fasting blood glucose levels and insulin tolerance. Conversely, the antagonistic antibody XMetD is currently in clinical trials for hypoglycemia treatment, while IRAB-B shows promise as a more cost-effective and rapid inducer of insulin resistance [49] (Table 1). This study not only elucidates a novel mechanism of hepatocarcinogenesis but also proposes a potential therapeutic strategy for liver cancer, emphasizing the value of targeting IR nuclear translocation in cancer treatment.

## Conclusions and recommendations

The ability of proteins to shuttle between the nucleus and cytoplasm is fundamental to numerous essential cellular processes and tissue- and cell type-specific functions. Many proteins necessitate transportation to the nucleus post-synthesis to execute their biological regulatory roles, particularly noteworthy is the nucleocytoplasmic shuttling of specific proteins like tumor suppressors and oncogenic proteins, whose abnormal nuclear localization often leads to cellular transformation or cancer progression. Membrane-bound transcription factors (MTFs) represent a distinct category of transcription factors that traverse from the cytoplasm to the nucleus through diverse nuclear entry pathways, where they engage with DNA and serve as transcriptional regulators to modulate the expression of target genes. These MTFs translocate into the nucleus either in their full-length form or as protein hydrolysis cleavage fragments [120]. Presently, the nuclear translocation modes of MTFs primarily encompass classical and non-classical pathways, albeit individualized specialized translocation routes have also been delineated. Moreover, each MTF exhibits distinct characteristics, thereby necessitating specific translocation modes for individual MTFs.

The mechanisms governing nuclear entry vary for MTFs residing in different cellular compartments. MTFs located on the plasma membrane typically enter the nucleus through processes such as regulated intramembrane proteolysis (RIP), depalmitoylation, or retrograde transport from the Golgi to the endoplasmic reticulum. Conversely, MTFs situated on the endoplasmic reticulum membrane gain access to the nucleus through regulated ubiquitin/proteasome-dependent processing (RUP), self-proteolysis, splice-dependent mechanisms, or retrograde transport to the Golgi for degradation [8]. Consequently, despite the increasing scrutiny of various MTFs, a plethora of MTFs remain to be identified, necessitating further exploration into their nuclear entry mechanisms and biological functions.

MTFs ingress into the nucleus and modulate various biological functions including apoptosis, proliferation, migration, and more. Additionally, they serve as transcription factors, orchestrating the expression of corresponding genes. Furthermore, nuclear MTFs are intimately linked with the onset and progression of cancer. A large number of studies have shown that MTFs inhibitors can effectively inhibit the occurrence and development of cancer, and have been put into clinical use. Consequently, targeting nuclear MTFs for cancer therapy has emerged as a viable strategy. Given the role of nuclear EGFR in drug resistance and tumor malignancy, researchers have investigated strategies to reduce its nuclear translocation by inhibiting Akt and Src-induced EGFR phosphorylation. Inhibitors of Akt and Src have successfully suppressed nuclear EGFR accumulation [121]. Based on this principle, celecoxib has also been explored for its potential to inhibit EGFR nuclear translocation and enhance radiosensitivity [122]. Studies suggest that this approach may be effective in treating prostate and breast cancers with nuclear EGFR expression [123]. Additionally, the combination of the dual tyrosine kinase inhibitor AEE788 with celecoxib has been shown to prevent β-catenin accumulation in the nucleus of tumor cells, which is beneficial for the treatment of metastatic colorectal cancer [124]. The development of anti-nuclear IR antibodies offers a more cost-effective and rapid treatment method for diabetes [49, 125].

Overexpression of importins and exportins is frequently reported across various malignancies, affecting cell growth, viability, differentiation, drug resistance, and the tumor microenvironment. Abnormal expression of nuclear transport-related genes observed in breast cancer, head and neck tumors and hematologic malignancies underscores the potential of nuclear transport proteins as diagnostic biomarkers for cancers, with combinations of these protein family members providing the most predictive value [126–128]. The clinical approval of selinexor, a specific inhibitor of exportin-1 (XPO1), marks the first nuclear protein inhibitor, fully demonstrating the importance of nuclear transport pathways in cancer therapy [129]. Collectively, these studies underscore the potential of targeting MTF nuclear translocation or directly targeting nuclear MTFs as promising avenues for cancer therapy.

However, the precise mechanisms through which MTFs impact cancer remain elusive, and the potential for these interventions to disrupt the original functions of such proteins on the cell membrane, potentially leading to unforeseen side effects, remains uncertain. Hence, investigating the role of membrane-localized nuclear-cytoplasmic shuttling proteins in cancer can deepen our comprehension of tumor cell biology and furnish robust support for the advancement of novel cancer treatment modalities.

## Data Availability

No datasets were generated or analysed during the current study.
